# Strength Training Frequency and Athletic Performance in High School Girls Basketball Players

**DOI:** 10.5114/jhk/184042

**Published:** 2024-04-15

**Authors:** Erika Viramontes, J. Jay Dawes, Jared W. Coburn, Robert G. Lockie

**Affiliations:** 1 Center for Sport Performance, Department of Kinesiology, California State University, Fullerton, Fullerton, CA, USA.; 2 School of Kinesiology, Applied Health and Recreation, Oklahoma State University, Stillwater, OK, USA.; 3 Tactical Fitness and Nutrition Lab, Oklahoma State University, Stillwater, OK, USA.

**Keywords:** adolescents, change-of-direction speed, female athletes, power, resistance training

## Abstract

This study investigated the effects of a six-week strength training intervention on the physical fitness of female high school athletes, with a focus on training frequency. Twenty-three female high school basketball athletes were recruited and split into two groups: one group participated in strength training once per week (S1), while the other participated in two training sessions per week (S2). The groups were not random as training sessions were voluntary, and some participants were only able to train once per week. Participants were tested before and after the intervention, and the data included: age, body height, body mass, body fat percentage, grip strength, leg/back dynamometer (LBD) strength, a seated medicine ball throw (MBT), a vertical jump (VJ), 505 tests from each foot, 0–5, 0–10, and 0–20 sprint times, and multistage fitness test shuttles. Data were analyzed by a two (time) x two (group) repeated measures analysis of variance (ANOVA; p < 0.05). When significant F ratios were detected in any ANOVA calculations, post hoc pairwise comparisons were conducted using the Bonferroni adjustment procedure. There were significant main effects for time that indicated the following: increased body height, body mass, grip strength, LBD strength, MBT distance, and VJ height, faster 505 times, and slower 0–5 and 0–10 m sprint times (p ≤ 0.021). There were no significant time by group ANOVAs or between-group main effects. These performance changes occurred irrespective of training frequency. High school girls who participate in at least one strength training session per week can improve their strength (grip, LBD), power (MBT, VJ), and change-of-direction speed (505).

## Introduction

Organized sports participation can benefit youth development, including fewer behavior problems, increased educational achievement, and better psychosocial adjustment (Linver et al., 2009). High school sports participation is also associated with improved adult lifestyle habits and cardiovascular fitness (Angeli et al., 2017). What can supplement success in high school sports is the addition of resistance training in the preparation of athletes (Behm et al., 2008). Resistance training has been recommended for youth by various professional associations (Behm et al., 2008; Faigenbaum et al., 2009). When performed with proper technique, resistance training has the potential to decrease the incidence and severity of injuries and improve athletic performance in young athletes (Behm et al., 2008). Increases in muscular strength and endurance, bone mineral density, motor coordination, maintenance of a healthy body weight, decreased body fat, and decreased depression are all potential benefits of strength training (Barahona-Fuentes et al., 2021; Dahab and McCambridge, 2009; Krolikowska et al., 2023; Mikoƚajec et al., 2017; Papla et al., 2022; Spieszny and Zubik, 2018;).

To provide specific examples, a higher repetition-moderate load resistance training program in children from 5 to 12 years of age increased muscle strength and endurance (Faigenbaum et al., 1999). The training groups participated in twice-weekly resistance training programs, which included either a lower repetition-higher load intervention or a higher repetition-moderate load intervention, for eight weeks. There was a 31–41% increase in a one repetition-maximum (1RM) leg extension, and a significant increase in leg extension muscular endurance, for both exercise groups. Findings from another study with 18 soccer players aged 12–15 years suggested that the addition of a resistance training program, in accompaniment with soccer training, could increase upper- and lower-body strength, and aid in physical development (Christou et al., 2006). The players followed a soccer-specific training program five times per week, with two specific groups; one completing the soccer program, and the other participating in the soccer program with the addition of strength training. The 16-week strength program involved two sessions a week and was programmed for 10 exercises, at 2–3 sets of 8–15 repetitions, with a load of 55–80% 1RM. After the training period, the group who completed the strength training program showed superior improvement in upper- and lower-body maximal strength (measured by a 1RM bench press and leg press, respectively), vertical jump height, and 30-meter (m) sprint speed (Christou et al., 2006).

High school-level athletes, however, can have different experiences based on school they attend. The program budget can dictate the quality and quantity of high school sports coaches, training facilities, team and athlete expenses, and the caliber of strength and conditioning programs offered to student-athletes. Despite the Title IX Educational Amendment 1972, which prohibited sex discrimination in any education program or activity receiving federal financial assistance in the USA, there is an imbalance in what females are afforded in the sporting environment (Silvers-Granelli, 2021). This includes diminished access to exercise equipment, lower coaching standards, medical staffing, and strength and conditioning programs (Parsons et al., 2021; Silvers-Granelli, 2021). If a school does not have a designated certified strength and conditioning coach on staff, the responsibility of providing programs usually lies on the head coaches of their respective sport (Reynolds et al., 2012). The quality of strength and conditioning programs provided to students could be greatly affected in such situations.

Indeed, strength and conditioning programs at the high school level may not be implemented by someone who is educated in best practices. One survey conducted by Reynolds et al. (2012) with coaches of girls’ and boys’ baseball/softball, basketball and soccer, found that most of the strength and conditioning programs were delivered and executed by coaches without some sort of personal trainer or strength and conditioning certification. Despite only a select handful of coaches being considered “certified” strength and conditioning coaches, a large percentage were interested in obtaining certifications and enhanced education. This suggests that coaches are aware of their lack of formal knowledge and the need to learn. What could support teachers pursuing further education is research demonstrating the value of strength and conditioning programs provided by professionals trained specifically in strength and conditioning. This is important in situations where athletes may have limited time to train, and need to make the most of what time they have available for resistance training.

This study compared the effects of participation in a structured 6-week strength training program, provided by strength and conditioning coaches, on the physical fitness of female high school athletes. Part of the sample participated in one session per week, while the rest of the sample participated in two sessions per week. The intent of this study was to demonstrate the potential impact of certified and experienced strength and conditioning professionals in helping prepare athletes at the high school level. Furthermore, this study also aimed to investigate whether one or two training sessions per week created similar results. It was hypothesized that athletes participating in the strength training program twice per week would demonstrate greater fitness improvements than athletes who participated only once per week.

## Methods

### Participants

Participants were recruited from one high school located in southern California. A total of 27 female high school athletes were available for this study (age: 15.22 ± 0.95 years; body height: 162 ± 8.07 cm; body mass: 61.52 ± 14.23 kg). Inclusion criteria comprised being part of the girls’ high school basketball team and participation in both pre- and post-testing. There were no athletes above the age of 18. Thus, before testing took place, parents signed a consent form to allow their children to participate in this study. Student-athletes signed their respective assent forms on the day of their first testing session. The procedures used in this study were approved by the Institutional Review Board of California State University, Fullerton (approval code: HSR-19-20-511; approval date: 15 June 2020).

### Procedures

The study was conducted during March–May in the Spring semester, which was the basketball off-season. All tests were completed within a two-hour session at the high school in the afternoon. If participants could not attend the original pre- and post-testing session, they completed a make-up session within three days of the original date. Age was recorded prior to testing. Body height of each participant was measured via a stadiometer (Health O Meter, Ontario, Canada) in centimeters (cm). The final selection of other tests was determined in consultation with the high school coaches. Athletes were tested before and after their participation in the strength training intervention. The test order was determined based on which ones would be least fatiguing being completed first, to those that would be most taxing completed last (McGuigan, 2015), in addition to the logistics of the testing location at the high school.

### Body Mass and Body Fat Percentage

Electronic digital scales (Model HBF-510, Omron Healthcare, Kyoto, Japan), which include bioelectrical impedance analysis, were used to record body mass and body fat percentage (BF%). While it was difficult to control for hydration, participants were instructed to maintain their typical diet and fluid intake prior to testing. Protocol guidelines provided from the manufacturer were followed to record BF% (Vasold et al., 2019). The participant’s age, body height in cm, and sex were entered into the device. The participant was not wearing shoes or socks when on the scale. They stepped onto the scale with their feet positioned on the foot and heel electrodes before holding the display unit with both hands until their body mass was displayed on the screen. The display unit also had electrodes on the handles, and the hands were positioned on top of these electrodes. Once the participant’s feet and hands were positioned on the appropriate electrodes (eight in total), they were told to stand upright and extend their arms, so that they were parallel to the ground. Equations from the device provided measurements of their body mass and BF%.

### Grip Strength

Grip strength provides a measure of upper-body strength and has been used to assess strength in adolescents (Hager-Ross and Rosblad, 2002), and was measured via a dynamometer (Takei Scientific Instruments, Japan). Participants kept their testing arm by their side throughout the assessment and squeezed the handle as hard as possible for approximately 2–3 s. Three trials for each hand were recorded to the nearest kg, with the best trial for each hand summed together to provide the grip strength metric.

### Leg/Back Dynamometer

Leg and back strength were measured using a leg/back dynamometer (LBD) (Fabrication Enterprises, Inc., New York, USA). This test provides a valid measure of lower-body strength and can be performed safely by those with a limited training background (Dawes et al., 2017). The participant was positioned so that her arms were extended and both hands were on the handle positioned at the mid-thigh (knee flexion angle of ~110°). From here, participants pulled up on the handle as hard as possible by attempting to extend the hips and knees. Three attempts were given, with the best trial being analyzed.

### Seated Medicine Ball Throw

Upper-body power was measured with a seated medicine ball-throw (MBT) (Lockie et al., 2018). Participants sat against a wall and projected a 4.5-kg medicine ball (Champion Barbell, Texas, USA) as far forwards as possible using a two-handed chest pass. The measurement was taken using a standard tape measure and was the perpendicular distance from the wall to where the ball contacted the ground. Three trials were completed, with a between-trials recovery time of ~60 s. The best trial was analyzed.

### Vertical Jump

The vertical jump (VJ) test was used to indirectly measure lower-body power (Lockie et al., 2014a, 2014b), and a jump mat (Just Jump, Probotics Inc., Huntsville) measured performance (McFarland et al., 2016). The participant stood on the mat keeping her heels on the floor, before completing a countermovement with an arm swing and jumping as high as possible. Participants were instructed to maintain straight legs during the flight, before landing on both feet with flexion of the hips, knees, and ankles. Within the software for the mat, jump height was calculated in inches and converted to cm. Each participant completed three trials, with ~60 s between trials, with the best trial used for analysis.

### 20-m Sprint

A 20-m sprint measured maximal running speed with procedures adapted from previous research (Post et al., 2022). Timing gates (Dashr Motion Performance Systems, Nebraska, USA) were positioned at 0 m, 5 m, 10 m, and 20 m, to measure the 0–5 m, 0–10 m, and 0–20 m intervals. Gate height was set at 0.93 m, and gates were positioned 2.4 m apart. Participants began each sprint from a standing start 50 cm behind the start line to trigger the first gate. Three trials were completed with time recorded to the nearest 0.01 s, and the best 20-m sprint trial was analyzed.

### 505 Change-of-Direction Speed Test

The 505 test measured change-of-direction (COD) speed (Post et al., 2022). This test required the participant to sprint 10 m past a timing gate (Dashr Motion Performance Systems, Nebraska, USA), then keep sprinting for another 5 m, before planting her foot on the turning line (15 m from the start line), turning back 180° to run past the timing gate. Participants completed three trials per foot (e.g., left-foot plant and turn, and right-foot plant and turn). For consistency, all participants planted with their left foot first for three trials, then planted with their right foot for three trials. The best trial for each foot was used for analysis.

### Multistage Fitness Test

The multistage fitness test (MSFT) measured maximal aerobic capacity (Lockie et al., 2020). Participants ran back and forth in an indoor gym, between two lines spaced 20 m apart indicated and marked on the ground with tape and cones. Running speed was standardized by pre-recorded auditory cues (i.e., beeps) played from an iPad handheld device (Apple Inc., Cupertino, California) connected via Bluetooth to a portable speaker (JBL FLIP 5, Los Angeles, CA), which was placed in the center of the running area. The test was terminated when the participant was unable to reach the lines twice in a row in accordance with the auditory cues, or by voluntary exhaustion. This test was scored according to the final stage and level the participant, which were used to calculate the total number of completed shuttles.

### Resistance Training Program

The training program was designed specifically to focus on muscular strength and hypertrophy. Programming guidelines for high school athletes were followed (Faigenbaum et al., 2009; Sheppard and Triplett, 2016). The program was designed with the team’s availability in mind (i.e., two sessions per week). Training sessions were not mandatory and were offered from 4–6 pm twice a week, on Mondays and Fridays. Participants could attend either one or both sessions, and could attend any training day (i.e., Monday or Friday). However, there were participants who could only attend one session per week because of outside commitments (e.g., club basketball), and the day participants did attend may have varied (i.e., some could only attend Monday, others only on Friday). This meant the exact load experienced by participants across their training was difficult, if not impossible, to track. Nevertheless, attendance was noted for all participants who completed the training program. Participants were placed into groups after the intervention based on their attendance and were not randomly allocated. Two coaches contributed to the prescription of the strength and conditioning program, and there was one coach at every training session. One of the coaches had obtained their bachelor’s degree in Kinesiology and was currently pursuing a master’s degree in Kinesiology as well. They also had their National Strength and Conditioning Association Certified Strength and Conditioning Specialist (CSCS) certification and had two years of experience coaching high school athletes and adults. The second coach had a bachelor’s degree in Kinesiology, and more than five years of experience coaching high school athletes.

Tables 1 and 2 below show the 6-week training program, including the warm-up and working sets. Abdominal-strengthening exercises were also programmed, with one of two regimens being completed after each session (Table 2). All participants completed the same program, which was designed to use minimal equipment to accommodate for large groups training simultaneously in a small weight room, which is the reality for many high schools. Although participants did not have their maximal strength measured for all exercises, loading was manipulated with respect to the target repetitions and abilities of the participants. It should be noted that participants completed basketball-related activities each week, which included scrimmages and sport-specific practice sessions. These were not controlled by the researchers and were not recorded as they varied across all the group.

**Table 1:** Weeks 1–3 of the resistance training program. s: seconds.

Day 1 (Monday)	Sets x Repetitions	Day 2 (Friday)	Sets x Repetitions
Week 1			
Warm-up		Warm-up	
Trap Bar Squat Jumps	3 x 10	Straight Arm Toss	2 x 10
Mini-Band Hip Extensions	3 x 10	1-2-3 Tempo Throws	2 x 10
Single-Leg Plate Jumps	3 x 10	Med-Ball Slams	2 x 10
		Eccentric Anchor Bar Punches	2 x 6
Working Sets		Working Sets	
A1. Barbell Romanian Deadlift	3 x 10	A1. Front Squat	1 x 10
A2. Single Leg Dumbbell Hip Thruster	3 x 10	A2. Eccentric Goblet Squat	4 x 8
A3. Mini-Band Internal and External Hip	3 x 10	B1. Incline Dumbbell Row	4 x 10
Rotation		B2. Eccentric Cable Row	4 x 10
B1. Decline Dumbbell Bench	4 x 10	C1. Band Face Pulls	4 x 10
B2. Overhead Cable Triceps Extension	4 x 10	C2. Incline Dumbbell Curls	4 x 10
B3. Band Triceps	4 x 10	C3. Eccentric Pronated Dumbbell Curls	4 x 5
Week 2			
Warm-up		Warm-up	
Dumbbell Squat Jumps	3 x 10	Dumbbell Step Back to Knee Ups	3 x 10
Band-Assisted Jumps	3 x 10	Barbell Squat Jumps	3 x 10
Plate Goblet Squat	3 x 10		
Working Sets		Working Sets	
A1. Dumbbell Snatch	3 x 10	A1. Back Squat	1 x 10
B1. Dumbbell Thrusters	3 x 8	A2. Eccentric Back Squat	3 x 8
B2. Bulgarian Split-Squats	3 x 12	B1. Dumbbell Row	4 x 10
C1. Dumbbell Shoulder Press	3 x 8	B2. Wide-Grip Band Lat Pulldown	4 x 10
C2. Halos with Plate	3 x 10	B3. Inverted Row	4 x 8
C3. Incline Dumbbell Triceps Extension	3 x 10	C1. Incline Barbell Bicep Holds	3 x 10–30 s
		C2. Band Bicep Curls	3 x 10
Week 3			
Warm-up		Warm-up	
Med Ball Lateral Slams	3 x 10	Trap Bar Squat Jumps	3 x 10
Anchor Bar Split Punches	3 x 10	Mini-Band Hip Extensions	3 x 10
Dumbbell Plate Jumps	3 x 10	Single-Leg Plate Jumps	3 x 10
Working Sets		Working Sets	
A1. Banded Goblet Squats	4 x 10	A1. Barbell Romanian Deadlift	3 x 10
A2. Banded Lateral Walks	4 x 8	A2. Single Leg Dumbbell Hip Thruster	3 x 10
B1. Dumbbell Bench	4 x 10	A3. Mini-Band Internal and External	3 x 10
B2. Dumbbell Chest Fly	4 x 10	Hip Rotation	
C1. Incline Barbell Triceps Hold	3 x 10–30 s	B1. Decline Dumbbell Bench	4 x 10
C2. Bench Push-Ups	3 x 10	B2. Overhead Cable Triceps Extension	4 x 10
		B3. Band Triceps	4 x 10

**Table 2:** Weeks 4–6 of the resistance training program and abdominal/core exercises completed every Monday or Friday session. s: seconds.

Day 1 (Monday)	Sets x Repetitions	Day 2 (Friday)	Sets x Repetitions
Week 4			
Warm-up		Warm-up	
Straight Arm Toss	2 x 10	Dumbbell Squat Jumps	3 x 10
1-2-3 Tempo Throws	2 x 10	Band-Assisted Jumps	3 x 10
Med Ball Slams	2 x 10	Plate Goblet Squat	3 x 10
Eccentric Anchor Bar Punches	2 x 6		
Working Sets		Working Sets	
A1. Front Squat	1 x 10	A1. Dumbbell Snatch	3 x 10
A2. Eccentric Goblet Squat	4 x 8	B1. Dumbbell Thrusters	3 x 8
B1. Incline Dumbbell Row	4 x 10	B2. Bulgarian Split-Squats	3 x 12
B2. Eccentric Cable Row	4 x 10	C1. Dumbbell Shoulder Press	3 x 8
C1. Band Face Pulls	4 x 10	C2. Halos with Plate	3 x 10
C2. Incline Dumbbell Curls	4 x 10	C3. Incline Dumbbell Triceps Extension	3 x 10
C3. Eccentric Pronated Dumbbell Curls	4 x 5		
Week 5			
Warm-up		Warm-up	
Dumbbell Step Back to Knee Ups	3 x 10	Med Ball Lateral Slams	3 x 10
Barbell Squat Jumps	3 x 10	Anchor Bar Split Punches	3 x 10
		Dumbbell Plate Jumps	3 x 10
Working Sets		Working Sets	
A1. Back Squat	1 x 10	A1. Banded Goblet Squats	4 x 10
A2. Eccentric Back Squat	3 x 8	A2. Banded Lateral Walks	4 x 8
B1. Dumbbell Row	4 x 10	B1. Dumbbell Bench	4 x 10
B2. Wide-Grip Band Lat Pulldown	4 x 10	B2. Dumbbell Chest Fly	4 x 10
B3. Inverted Row	4 x 8	C1. Incline Barbell Triceps Hold	3 x 10–30 s
C1. Incline Barbell Bicep Holds	3 x 10–30 s	C2. Bench Push-Ups	3 x 10
C2. Band Bicep Curls	3 x 10		
Week 6			
Warm-up		Warm-up	
Trap Bar Squat Jumps	3 x 10	Straight Arm Toss	2 x 10
Mini-Band Hip Extensions	3 x 10	1-2-3 Tempo Throws	2 x 10
Single-Leg Plate Jumps	3 x 10	Med Ball Slams	2 x 10
		Eccentric Anchor Bar Punches	2 x 6
Working Sets		Working Sets	
A1. Barbell Romanian Deadlift	3 x 10	A1. Front Squat	1 x 10
A2. Single-Leg Dumbbell Hip Thruster	3 x 10	A2. Eccentric Goblet Squat	4 x 8
A3. Mini-Band Internal and External Hip	3 x 10	B1. Incline Dumbbell Row	4 x 10
Rotation		B2. Eccentric Cable Row	4 x 10
B1. Decline Dumbbell Bench	4 x 10	C1. Band Face Pulls	4 x 10
B2. Overhead Cable Triceps Extension	4 x 10	C2. Incline Dumbbell Curls	4 x 10
B3. Band Triceps	4 x 10	C3. Eccentric Pronated Dumbbell Curls	4 x 5
Abdominal/Core Exercises			
Hanging Straight Leg Lifts	3 x 30–60 s	Banded Dead Bug	3 x 30–60 s
Straight Leg Holds	3 x 10	Straight Leg Holds	3 x 30–60 s
Russian Twists	3 x 10	Leg Lifts	3 x 10
Toe Touches	3 x 10	Alternating Eccentric Leg Taps	3 x 10

### Statistical Analysis

All statistical analyses were computed using the Statistics Package for Social Sciences (version 28.0; IBM Corporation, NY, USA). Descriptive statistics (mean ± standard deviation [SD]) were calculated for each variable. The sample was split into two groups: those that completed one training session per week (S1) and those who completed two sessions per week (S2). Data were analyzed via a two (time; pre and post) by two (S1 and S2) repeated measures analysis of variance (ANOVA) (*p* < 0.05). As only two repeated measures were employed, the assumption of Mauchly’s test of sphericity was not applicable (Lockie et al., 2014a, 2014b). All other repeated measures ANOVA assumptions were considered, with the Levene’s statistic used to determine homogeneity of variance. When a significant *F* ratio was detected, post hoc tests were performed using the Bonferroni adjustment procedure.

## Results

Twenty-three participants met the inclusion criteria (Table 3). Eight athletes attended one training session per week (S1), and 15 athletes attended two sessions per week (S2). For body height, the main effect of time was significant (*F*_(1,21)_ = 6.228, *p* = 0.021, ηp^2^ = 0.229), as participants got taller over the course of the study. The time by group ANOVA (*F*_(1,21)_ = 0.009, *p* = 0.923, ηp^2^ < 0.001) and main effect between groups (*F*_(1,21)_ = 0.131, *p* = 0.721, ηp^2^ = 0.006) were not significant. The main effect of time was significant for body mass (*F*_(1,21)_=23.363, *p* < 0.001, ηp^2^ = 0.527); participants were heavier after the six weeks. The time by group ANOVA (*F*_(1,21)_ = 0.175, *p* = 0.680, ηp^2^ = 0.008) and main effect between groups (*F*_(1,21)_ = 0.004, *p* = 0.949, ηp^2^<0.001) were not significant. One participant was excluded from BF% analysis due to not being able to remove her socks for religious reasons. The main effect of time (*F*_(1,19)_ = 0.850, *p* = 0.368, ηp^2^ = 0.043), time by group ANOVA (*F*_(1,19)_ = 0.324, *p* = 0.576, ηp^2^ = 0.017), and main effect between groups (*F*_(1,19)_ = 0.649, *p* = 0.430, ηp^2^ = 0.033) were not significant.

**Table 3:** Descriptive data (mean ± SD) for pre- and post-test results (age, height, body mass, body fat percentage [BF%], grip strength, leg/back dynamometer [LBD] strength, medicine ball throw [MBT], vertical jump [VJ], 0–5 m, 0–10 m, and 0–20 m intervals from a 20-m sprint, 505 change-of-direction speed tests from the left and right feet, and multistage fitness test [MSFT] shuttles) between participants who trained once (S1) or twice (S2) per week over six weeks.

	S1 (n = 8)		S2 (n = 15)	
	Pre	Post	Pre	Post
Age	15.50 ± 1.07	15.63 ± 1.19	14.93 ± 0.88	15.07 ± 0.96
Height (cm)	163.0 ± 6.84	163.46 ± 7.24*	161.57 ± 9.67	162.07 ± 9.70*
Body Mass (kg)	62.08 ± 15.08	63.21 ± 15.97*	62.41 ± 15.83	63.77 ± 16.16*
BF%	33.11 ± 9.11	32.60 ± 9.51	35.65 ± 6.49	35.53 ± 6.03
Grip Strength (kg)	57.25 ± 13.04	63.63 ± 12.94*	57.07 ± 9.42	62.57 ± 9.00*
LBD (kg)	64.25 ± 30.77	101.5 ± 20.81*	61.07 ± 19.87	96.0 ± 11.77*
MBT (m)	3.56 ± 0.60	4.23 ± 0.53*	3.56 ± 0.41	4.44 ± 0.44*
VJ (cm)	39.88 ± 6.74	43.94 ± 6.19*	39.57 ± 5.80	45.21 ± 6.23*
0–5 m (s)	1.10 ± 0.07	1.26 ± 0.17*	1.12 ± 0.13	1.37 ± 0.33*
0–10 m (s)	2.00 ± 0.09	2.13 ± 0.20*	2.03 ± 0.20	2.24 ± 0.34*
0–20 m (s)	3.64 ± 0.14	3.70 ± 0.28	3.64 ± 0.32	3.83 ± 0.43
505 Left (s)	2.90 ± 0.23	2.69 ± 0.18*	2.92 ± 0.17	2.71 ± 0.18*
505 Right (s)	2.86 ± 0.18	2.65 ± 0.23*	2.88 ± 0.24	2.68 ± 0.18*
MSFT (shuttles)	44.83 ± 10.63	51.67 ± 13.53	44.85 ± 14.05	50.08 ± 12.43

* Significantly (*p* < 0.05) different from pre-test.

The main effect of time was significant for grip strength (*F*_(1,21)_ = 44.865, *p* < 0.001, ηp^2^ = 0.681); participants were stronger after training. The time by group ANOVA (*F*_(1,21)_ = 0.244, *p* = 0.627, ηp^2^ = 0.011) and main effect between groups (*F*_(1,21)_ = 0.019, *p* = 0.893, ηp^2^ = 0.001) were not significant. Similar results were observed for the LBD. The main effect of time was significant (*F*_(1,21)_ = 77.142, *p* < 0.001, ηp^2^ = 0.786), while the time by group ANOVA (*F*_(1,21)_ = 0.079, *p* = 0.781, ηp^2^ = 0.004) and main effect between groups (*F*_(1,21)_ = 0.308, *p* = 0.585, ηp^2^ = 0.014) were not. Participants also improved their power, as measured by the MBT and VJ, following the intervention, with no differences between the S1 and S2 groups. Specifically, the main effect of time was significant for both the MBT (*F*_(1,21)_ = 90.184, *p* < 0.001, ηp^2^ = 0.881) and VJ (*F*_(1,21)_ = 85.248, *p* < 0.001, ηp^2^ = 0.802). However, time by group ANOVA (MBT: *F*_(1,21)_ = 1.667, *p* = 0.211, ηp^2^ = 0.074; VJ: *F*_(1,21)_ = 2.249, *p* = 0.149, ηp^2^ = 0.097) and main effect between groups (MBT: *F*_(1,21)_ = 0.298, *p* = 0.591, ηp^2^ = 0.014; VJ: *F*_(1,21)_ = 0.033, *p* = 0.857, ηp^2^ = 0.002) were not.

For the 20-m sprint, there was a significant effect for time for the 0–5 m (*F*_(1,21)_ = 9.387, *p* = 0.006, ηp^2^ = 0.309) and 0–10 m (*F*_(1,21)_ = 7.157, *p* = 0.014, ηp^2^ = 0.254) intervals, which indicated that after training participants were slower. However, the main effect for time was not significant for the 0–20 m interval (*F*_(1,21)_ = 3.888, *p* = 0.062, ηp^2^ = 0.156). The time by group ANOVA (0–5 m: *F*_(1,21)_ = 0.603, *p* = 0.446, ηp^2^ = 0.028; 0–10 m: *F*_(1,21)_ = 0.376, *p* = 0.546, ηp^2^ = 0.018; 0–20 m: *F*_(1,21)_ = 0.984, *p* = 0.332, ηp^2^ = 0.045) and main effect between groups (0–5 m: *F*_(1,21)_ = 0.899, *p* = 0.354, ηp^2^ = 0.041; 0–10 m: *F*_(1,21)_ = 0.619, *p* = 0.440, ηp^2^ = 0.029; 0–20 m: *F*_(1,21)_ = 0.247, *p* = 0.625, ηp^2^=0.012) were not significant for all sprint intervals. There were significant main effects for time for both the left- (*F*_(1,21)_ = 36.308, *p* < 0.001, ηp^2^ = 0.634) and right-foot (*F*_(1,21)_ = 33.403, *p* < 0.001, ηp^2^ = 0.614) 505 tests. The time by group ANOVA (Left: *F*_(1,21)_ < 0.001, *p* = 0.986, ηp^2^ < 0.001; Right: *F*_(1,21)_ = 0.056, *p* = 0.816, ηp^2^ = 0.003) and main effect between groups (Left: *F*_(1,21)_ = 0.058, *p* = 0.811, ηp^2^ = 0.003; Right: *F*_(1,21)_ = 0.126, *p* = 0.726, ηp^2^ = 0.006) were not significant for the 505 from either foot. Participants improved COD speed, which was indicated by faster 505 times from each foot, regardless of the group. Lastly, for the MSFT, the main effects for time (*F*_(1,21)_ = 3.866, *p* = 0.066, ηp^2^ = 0.185), time by group ANOVA (*F*_(1,21)_ = 0.068, *p* = 0.797, ηp^2^ = 0.004), and main effect between groups (*F*_(1,21)_ = 0.020, *p* = 0.890, ηp^2^ = 0.001) were all non-significant.

## Discussion

This study investigated the effects of a strength training program, designed by certified strength and conditioning coaches, with regards to the frequency of training in high school girls basketball players. It was expected that participants who participated in two sessions per week would present better results than those who only participated once per week. However, this was not the case; most test results indicated similar changes in both the S1 and S2 groups. This was the first exposure to strength training for most of the athletes on this girls’ basketball team, as similar to other schools, most of the girls’ teams on campus had very limited access to the weight room. The study results may have reflected this, as both groups experienced comparable adaptations, regardless of training frequency. Nonetheless, the results were generally positive which indicates how structured strength training, even performed once per week, can be beneficial for high school female athletes.

There was an increase in body height and mass for both groups, with no significant between-group differences. Maturation was not considered within this study, thus it should be noted that the body growth of participants could influence the results (Yapici et al., 2022). Nevertheless, specific to body mass, since BF% from pre- to post-testing did not change, it is possible that there was an increase in lean body mass. However, there may have been a concurrent increase in fat and lean body mass with growth of the athletes, which could also explain why BF% did not change. Nonetheless, past research has also shown favorable changes in muscle quality for adolescents (Naimo and Gu, 2022), and bone mineral density in adolescent females (Nichols et al., 2001), can occur as a result of the participation in resistance training. The results from the current study would suggest that 1–2 resistance training sessions per week may have positively impacted body composition in female high school athletes.

Strength measured by grip strength and the LBD increased following the intervention, regardless of training frequency. The same was true for the power measures of the MBT and VJ. These results were expected as there was an emphasis placed on building strength within the prescribed program. Even a relatively small amount of stimulus (i.e., 1–2 sessions per week) was enough to create beneficial strength adaptations, which is similar to past research findings (Faigenbaum et al., 2002; Naimo and Gu, 2022; Wakely et al., 2022). Previous research has also shown how strength training interventions can improve muscular power in adolescent athletes (Slimani et al., 2018; Wakely et al., 2022). Although there was an increase in muscular strength, it is important to note that adolescents may still be growing, and the strength and power increases could have been influenced by the process of maturation within this age group (Yapici et al., 2022). Nonetheless, given the ~10–11% increases in grip strength, ~57–58% increases in LBD scores, ~19–25% increases in MBT distance, and 10–14% increases in VJ across both groups, it could be expected that the intervention was successful in improving strength and power of the participants.

Regardless of training frequency, there were significant increases in 0–5 m and 0–10 m sprint times, which indicated that the participants were slower after the intervention. There was no significant change for the 0–20 m sprint interval. Sprinting over short (~5 m) and longer distances (~20 m) could be considered separate and specific biomechanical and neuromuscular actions (Harris et al., 2008). This may have contributed to the non-significant change in 20-m sprint time. Nevertheless, the slower 0–5 m and 0–10 m intervals, indicative of initial acceleration (Lockie et al., 2011), could be due to several factors; the participant’s increased body mass (i.e., greater inertia), residual fatigue from the strength training program, or participants not having enough training specific to linear speed. It should be reinforced that the intervention occurred during the off-season, and in a typical periodization model, linear speed training should take on greater focus closer towards the competition season (Haff, 2016). Previous research has shown that measures of strength (3RM squat) (Cronin and Hansen, 2005), and lower-body power (VJ height) (Banda et al., 2019), were not significantly correlated with speed over 20–30 m. Improving strength and jumping ability may not be sufficient to enhance 0–20 m sprint performance in high school girls. As there was no specific linear speed training to address sprinting technique, this could have contributed to the 0–20 m sprint data seen in this study.

In contrast to linear speed, COD speed, as measured by 505 times, was significantly faster following the intervention for both the S1 and S2 groups. Relationships between measures of strength and COD performance in female athletes have been demonstrated in past research (Vescovi and McGuigan, 2008). Moreover, Spiteri et al. (2013) detailed that individuals with greater lower-body strength produced greater ground reaction force during a plant and cut, and thus, were able to modify their lower-body positioning while producing faster COD times. Due to the increases in lower-body strength observed by participants in this study, it is possible that this helped improve 505 times. Research suggests coaches should aim to develop a well-rounded strength base in athletes, ensuring eccentric strength is developed as effectively as possible (Spiteri et al., 2014), in order to obtain improvements in COD ability. The inclusion of numerous exercises that emphasized eccentric work in the training program (e.g., eccentric back squats, rows, and banded work), likely influenced the COD results seen for both groups. The positive COD speed adaptations occurred regardless of whether participants completed one or two strength training sessions per week.

Aerobic fitness is crucial in basketball (Ben Abdelkrim et al., 2010). Nonetheless, the current results demonstrated that there were no significant changes in the number of MSFT shuttles completed following the intervention, regardless of training frequency. This was not unexpected, as aerobic capacity was not emphasized within the strength training program. Although participants in this study did participate in scrimmages throughout the 6-week intervention as part of their basketball practice, general conditioning has greater relevance closer to the season and was not a focus of basketball training for participants at this point in their season (Haff, 2016). Nonetheless, even with the gains in body mass and muscular strength for both the S1 and S2 groups, aerobic fitness as measured by the MSFT did not get worse, which is a net positive for the intervention.

There are some study limitations that should be noted. The true amount (i.e., training sessions per week) and type (e.g., sport-specific practice and skills development, resistance training) of training were difficult to track. High school athletes often compete and train for several sports and may perform additional personal training as well. However, it is not realistic to control all outside physical activity for competitive high school athletes. More training sessions could have influenced the results (e.g., VJ, linear and COD speed), but since they were not all tracked outside of what was done at the school, they cannot be directly linked to any performance improvements. The number of participants was relatively small, as the study used a convenience sample, thus the participants’ number was limited. Nevertheless, the sample size was comparable to other studies investigating high school-aged populations (Christou et al., 2006; Secomb et al., 2017). The study did not have a control group, although other training studies have not used a control group either (Lockie et al., 2014a, 2014b; Secomb et al., 2017). No control group often applies in situations where it is not feasible to not provide training opportunities to athletes, such as in this study. Nonetheless, completing basketball training alone could still have influenced the fitness level of participants. There was not a randomized participant assignment, since they were placed into groups (i.e., S1 and S2) based on their choice of attended sessions. This could have resulted in load differences between participants and groups. The attendance of the study participants is also a practical reality in the high school environment, thus the results are reflective of a real-world situation for these adolescent groups.

## Conclusions

The results showed significant improvements in participants following strength training in body mass, grip strength, LBD, MBT, VJ, and 505 times in both the S1 and S2 groups. It should be acknowledged that there were increases in 0–5 m and 0–10 m sprint times, and no significant changes for the 0–20 m sprint interval and MSFT. However, these variables would be targeted later in the cycle of a properly periodized strength and conditioning program. These data suggest a net positive outcome from completing a strength training program designed by certified strength and conditioning coaches, regardless of training frequency. Although more research is needed, this study supports implementing at least one strength training session per week to improve the athletic performance of high school girls.
